# Proximity and waiting times in choice models for outpatient cardiological visits in Italy

**DOI:** 10.1371/journal.pone.0203018

**Published:** 2018-08-30

**Authors:** Chiara Seghieri, Martina Calovi, Francesca Ferrè

**Affiliations:** 1 Laboratorio Management e Sanità, Institute of Management, Scuola Superiore Sant’Anna, Pisa, Italy; 2 Geoinformatics and Earth Observation Laboratory, Department of Geography and Institute for CyberScience, The Pennsylvania State University, Pennsylvania, United States of America; Griffith University, AUSTRALIA

## Abstract

We apply mixed logit regression to investigate patients' choice of non-emergency outpatient cardiovascular specialists in Tuscany, Italy. We focused on the effects of travel time and waiting time. Results reveal that patients prefer clinics nearby and with shorter waiting times. Differences in patient choice depend on age and socioeconomic conditions, thus confirming equity concerns in the access of non-acute services. Our results could be used to optimize the allocation of resources, reduce inequities and increase the efficiency and responsiveness of outpatient systems considering patient preferences.

## Introduction

There are some instances where patients have a choice of healthcare providers for the diagnosis and treatment of their conditions, especially in systems where patients can access any provider and competition exists among providers or third party payers. Several high-income countries have introduced market-based health reforms and policies that have focused on increasing user choice by promoting competition and therefore allowing mobility across health care providers [1]. The aim was to increase service capacity, enhance efficiency, improve the quality of the health care delivered [2–5] and enhance patient empowerment [6]. For example, since 2008 the UK has invested in the expansion of patient choice and has encouraged hospitals to compete within a pseudo-market with fixed prices aimed at reducing spending and improving health system performance [7].

Provider selection can be based on a heterogeneous set of information, which include the quality of services, standard of facilities and technologies, reputation and image of the provider [8], attitudes and behavior of personnel, prior experience and recommendations [9–12] and other factors such as affordability (costs), accessibility in terms of both physical accessibility (proximity) and adequacy of service supply in relation to population.

This study considers how travel time and waiting times influence the patients’ choice of outpatient services in the regional healthcare system in Tuscany (Italy).

The analysis is not confined to the traditional regional model where the key variable is to understand whether or not patients receive treatment in the administrative region they reside in [13], but analyzes patient mobility within a health care regional system at a micro-level. The selection of the outpatient setting is important because of the centrality that the outpatient system plays in meeting the population’s needs at a more local level, especially with regard to access to specialist consultations and the appropriateness of such setting for non-acute cardiovascular care.

We investigated patients’ choice of a public outpatient clinic for non-emergency cardiological visits considering travel time (often used as a proxy of distance) and waiting time, while taking into account the patients' demographic and socioeconomic characteristics (age, sex, migrant status and income level) to incorporate equity expectations.

**Fig 1:**
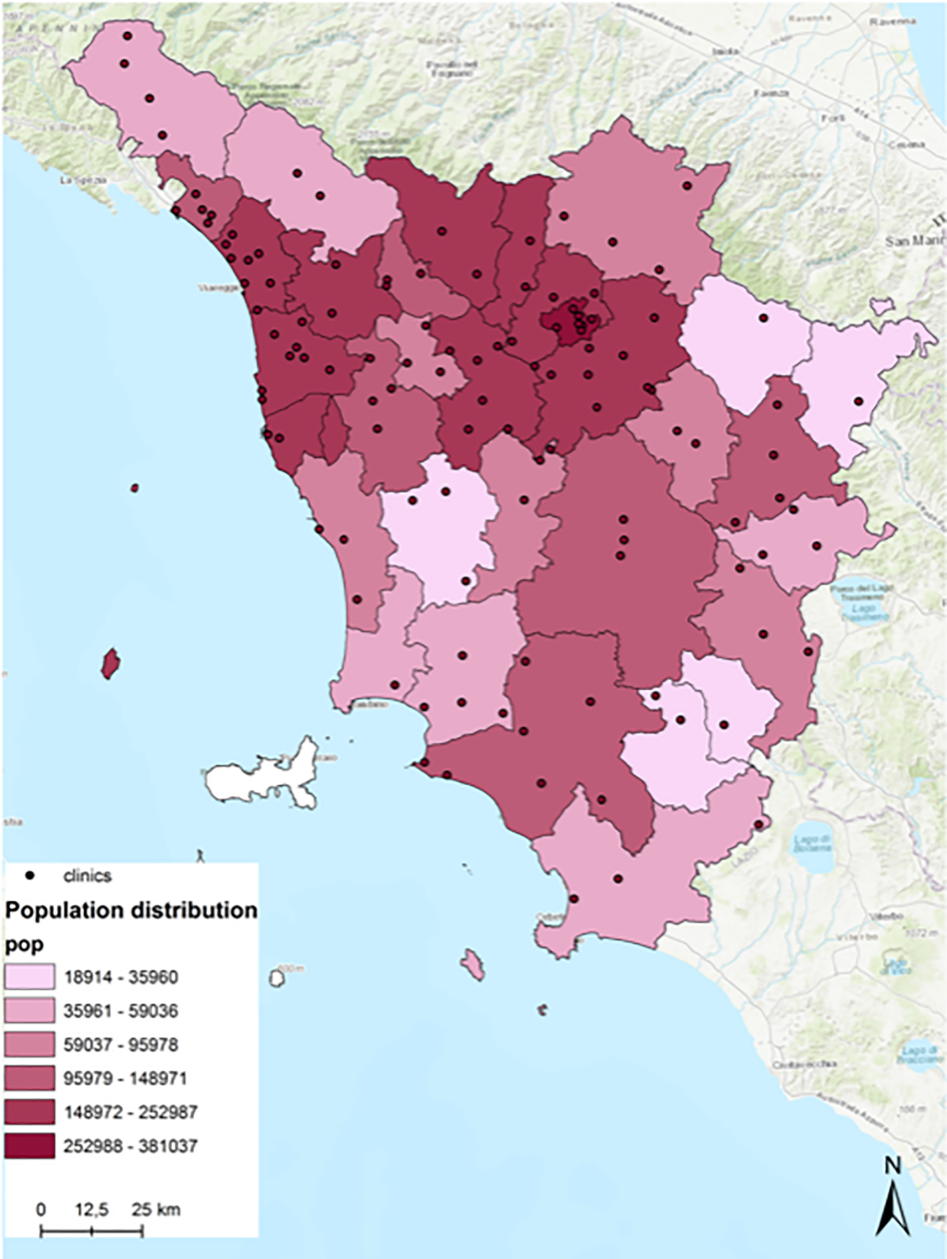
Distribution of population and public providers (outpatient clinics) by health districts in Tuscany 2016.

We selected cardiological visits because of: i) the high volume (11.50% of total regional outpatient visits in 2016) in fact in Tuscany cardiovascular conditions are the second cause of death after cancer [14]; ii) the significant number of outpatient clinics across the region (121 outpatient clinics registered at least 30 cardiologic visits in 2016) (Fig 1); and iii) the relatively high mobility of patients across health districts (almost one out of four patients travels outside their district of residence). Since Italian patients can choose their preferred provider in both hospital and non-urgent care, understanding the factors that facilitate or hinder patient mobility is fundamental [13]. Understanding patient mobility (willingness to travel) may be of interest to policymakers in order to ensure equity of access to NHS services and to adequate plan service capacity. It should thus support efficient allocation of the resources without affecting the quality of services provided to the population.

In order to make inferences on the study of choice behavior, we used a mixed logit regression to identify which of the considered spatial and non-spatial variables most affected the patients’ choices.

The paper is structured as followed: the background outlines a literature review on patients’ choices. The methodology and the data used are described followed by the results of the analysis. The paper ends with the discussion and conclusions.

## Background

The literature suggests that patients use several inputs when choosing a healthcare provider. Most of the evidence relates to hospital choices, highlighting how decision inputs vary depending on the urgency of the situation (in emergency conditions, physicians, ambulances and the distance to services play an active part), the type of services needed, and the configuration of the healthcare market [13,15,16].

The review by Aggarwall et al. (2016) on patient mobility for elective surgery summarizes the empirical evidence from countries that enable patients to choose their health care provider [15]. It should be noted that patient mobility involves three distinct categories: mobility due to patient preference; mobility due to the physician’s advice, and mobility due to an insufficient local supply. When investigating patient choice, the choice influenced by the physicians are difficult to discern from the choices taken exclusively by the patient [15].

In studies assessing the relative impact of patient-service interaction with health care services, the important dimensions to be considered are acceptability, affordability, availability, physical accessibility and accommodation [17]. The provision of choice and the likely increase in travel time or distance influence the access to care [16]. Thus, it is expected that patients and their caregivers, need to make a trade-off between these dimensions. Patients usually select the best treatment that they think the health system can provide, or the fastest service available (i.e. the provider with the lowest waiting time) or the nearest service (i.e. proximity to where they live). It should be noted that waiting times are often not directly affected by volume or capacity but rather a mix of system inefficiency, a lack of appropriateness in the prescription phase and a lack of professional resources [18].

For routine consultations, travel time, with the associated travel costs, can be an important driver in selecting providers (e.g., accessibility by public and private transport reduces mobility outside the reference region). The literature shows that one of the most important factors in hospital choice is the closeness of the hospital to the patients’ place of residence [10,12]. Also, research findings by Gesler and Meade suggest that the proximity to the patient's residence is an important determinant in the access and use of health services access and use, and may have a differential impact on people with different socio-demographic backgrounds [19]. In fact, patient choice may affect equity because of the different willingness to travel among socioeconomic groups [16].

Empirical studies report that older patients that belong to the lowest socioeconomic groups, and non-white patients are more likely to receive treatment from their nearest provider (either measured in terms of distance or travel time), or a hospital located within their region. Equity of access remains an issue, and it may be that hospitals within socioeconomically deprived areas with older demographic profiles have to manage far more complex patient cohorts (both medically and socially), which subsequently affects their quality outcomes. In contrast, shorter waiting times, higher hospital quality, and the availability of advanced technology increase the likelihood of patient mobility to seek care.

We believe that these factors also apply to outpatient care, with the difference that the appropriateness criteria for specialized medical care for outpatients (e.g., cardiology visits) are less clearly established compared to hospital procedures, and patient demand is more elastic in terms of the price (such as travel costs and co-payment) [20]. Overall, patient choice raises important questions about the way health care is delivered and accessed. The article provides evidence of patient preferences regarding outpatient services, focusing on the trade-offs and relationships between waiting time and distance to the outpatient clinic in different patient groups.

## Materials and methods

Studies on patient choice have predominantly used data derived from surveys, asking individuals about recent health care episodes or their responses to hypothetical scenarios, or they have used actual patient data from hospital or primary care episodes [15]. We ran a retrospective analysis using the administrative healthcare data of outpatient visits of residents in Tuscany for 2016. The administrative data were integrated into a GIS environment, in order to visualize the geographical distribution of the outpatient clinics.

Italy’s health-care system is a regionally based National Health Service (NHS), which provides universal coverage largely free of charge at the point of delivery [21]. Tuscany is a large region in central Italy characterized by a non-competitive health system where patients are free to choose any provider. The regional healthcare system comprises three local health authorities, four teaching hospitals and 34 health districts, which are in charge of the organization and delivery of services for local health networks, social care and social integration. Tuscany has a total population of over 3.7 million inhabitants (about 6.2% of the Italian population) (Demo Istat); high-density health districts are around the city of Florence with a population of over 360,000 inhabitants (Fig 1).

In Tuscany, mainly public providers deliver outpatient/walk-in care (in 2016 over 90% of all outpatient and laboratory services were provided either by public local health authorities or by public hospitals). Outpatient care is reimbursed using a tariff per unit of care (prospective payment system) and patients are asked to contribute to the cost via co-payments with exemptions based on gross family income, age, chronic and rare conditions and disabilities [21].

Using anonymized individual-level administrative outpatient care data in Tuscany from 2016, we extracted non-emergency cardiological visits for the adult resident population, characterizing each patient by socio-demographic data and place of residence. Data were anonymized at the Regional Health Information System Office where each patient was assigned a unique identifier that was the same for all administrative databases. This identifier does not disclose the patient’s identity and other sensitive data. The study was carried out in compliance with the Italian law on privacy, and approval by an Ethics Committee was not required. Patients were selected if they had had a cardiology visit within the reference year prescribed by the healthcare system in Tuscany, excluding direct access visits, pre and post-surgery, follow-up and screening. We considered cardiology visits listed with a non-urgent clinical priority i.e. a specialized visit needing a response either within 30 days (deferrable) or with no maximum waiting time, following the Ministry of Health guidelines on waiting times [22]. We excluded patients treated by outpatient public clinics who registered fewer than 30 visits per year and residents of the isle of Elba.

The selection criteria revealed 93,007 eligible patients and 121 public outpatient clinics throughout Tuscany. We only model the choice between public providers and did not include the choice to go private or to go to an outpatient clinic outside Tuscany.

Each patient was characterized by age (divided into 5-years categories), sex (male, female), income (we classified patients with incomes above and below 36,152 euros) and migrant status. Information on income classes is included in the outpatient database and is based on exemption to co-payment for specialist care (ref.: Decrees of the Regional Council n.722/2011, n.723/2011 and n.738/2011). Specifically in Tuscany, additional co-payments are not applied for patients with a yearly family income or Equity Economic Indicator (ISEE) below 36,151.98 euro (http://www.regione.toscana.it/cittadini/salute/ticket). Migrant status was dichotomized between patients from countries with high migratory pressure (Eastern Europe, Southeast Asia, and Latin America) versus others.

From the same administrative data source, information on waiting times was collected. Waiting times are defined by the number of days elapsing between the time the patient booked the visit and the day the patient received the outpatient service. From the individual level data, we computed the median waiting time distribution. The median was used as opposed to the mean in order to reduce the effect of outliers, which represent measurement or coding errors.

Each of the 121 clinics was integrated into a GIS environment, geolocating them (using their address) over the 34 health districts in Tuscany. On average, health districts have a resident population of 110,372 and about 3.5 outpatient clinics providing cardiologic visits.

To analyze the time patients travelled to health care services, studies on patient choice use the Euclidean/linear distance or travel time. For this study, the regional road network, available on the Open Toscana website (http://open.toscana.it/) was used to calculate the travel distances. Dasymetric mapping was used to identify the centroids of the patients. This gives the best estimates of the distribution of the aggregated population data within each unit of analysis, weighting the number of patients who live in the area over the real residential area [23,24]. This estimation was calculated by interpolating areal data in order to extract only the residential urban land use areas from the regional land use data obtained from the available online dataset. The travel times between the patients’ centroids (origins) and the outpatient clinics (destinations), were obtained by running an OD matrix, which returned 3,993 possible combinations.

Patient choice between outpatient clinics was modeled through a mixed logit model. The mixed logit model was deemed the most appropriate since it enables coefficients to vary between patients and thus to account for heterogeneity in preferences, and it also relaxes the assumption of independence from irrelevant alternatives [25]. Patient socio-demographic characteristics were included in the model as interactions with the attributes of the clinics: travel time and waiting time. Additionally, the cluster-robust standard error option was specified in the model to account for clustering at healthcare provider level.

All the statistical analyses were run using SAS version 9.4 (SAS Institute), the mixed logit regressions were run using user-written “mixlogit” STATA programme [26] and the geographical analyses were run using ArcMap version 10.3.1 (Esri).

## Results

In 2016, 121 outpatient clinics in Tuscany supplied on average 769 specialized cardiological visits, registering a minimum of 31 and a maximum of 3,318 visits.

On average, patients waited 37 days (SD = 52) to have a cardiology visit and the mean travel time to the chosen clinic was 27 minutes (SD = 21). Only 25.83% of the population sample chose the closest outpatient service which on average recorded a waiting time of 42 days.

Table 1 presents the descriptive statistics of the patient characteristics. The chosen variables characterize patients by age, sex, income level and migrant status (i.e. a patient from countries with strong migratory pressure, which includes Eastern Europe, Southeast Asia, and Latin America countries). The average age of patients was 67 years old, and 48% of the total sample were males. Less than 5% of patients resident in Tuscany were from countries with strong migratory pressure. As expected, more than 90% of patients had a family income of below 36,152 euros. This percentage is in line with the regional estimates provided by the Regional Agency for Health (ARS) and the Regional Institute for Economic Planning (IRPET), according to which around 80% of the population belong to this income class [27].

**Table 1:** Patient characteristics.

Variable	Obs	Mean	SD	Min	Max
**Patients’ age (year)**	93,007	67.27	15.32	18	100
**Men**	93,007	0.48	0.50	0	1
**Immigrant (patients originating from countries with high migratory pressure)**	93,007	0.04	0.20	0	1
**Low income (< 36,152 euro)**	93,007	0.92	0.27	0	1

Table 2 shows the results of the mixed logit regression run with model main effects and with interactions with individual socioeconomic variables.

**Table 2:** Mixed logit regression results.

	Model 1			Model 2		
Variable	Coeff.	SE	Sign.	Coeff.	SE	Sign.
**Median waiting time**	-0.015	0.002	***	-0.017	0.003	***
**Ln(Travel time)**	-2.269	0.021	***	-1.939	0.045	***
*Interaction terms*						
**Male x Ln(Travel time)**				0.018	0.035	ns
**Age x Ln(Travel time)**				-0.023	0.005	***
**Immigrant x Ln(Travel time)**				-0.052	0.034	ns
**Low income x Ln(Travel time)**				-0.165	0.030	***
**Male x Waiting time**				-0.001	0.002	ns
**Age x Waiting time**				0.001	0.000	ns
**Immigrant x Waiting time**				0.004	0.002	***
**Low income x Waiting time**				0.001	0.002	ns
**Standard deviation of individual heterogeneity specific to waiting times**	0.014	0.001	***	0.014	0.002	***
**Observations**	93,007			93,007		
**Log-likelihood**	24955.01			249251.73		

Note: The coefficient represents the mean relative utility of each attribute conditional on the other attribute while the standard deviation of the random coefficient reflects the degree of heterogeneity among patients in the utility of the given attribute.

Significance levels:

*** p ≤.001.

** p ≤ .01.

* p ≤.05.

ns > .05.

Model 1 includes only the travel time and waiting time. The main effects are statistically significant and are negative, meaning that patients preferred clinics nearby and with lower waiting times. A significant preference heterogeneity among patients was found only for the waiting time.

After the inclusion of patient sociodemographic characteristics (Model 2), the coefficients of the main effects are still significant, and the sign remains the same. In both models, travel time has more weight on a patient's decision to opt for one clinic compared to waiting time.

The elderly and patients in the lowest income class were more likely to choose clinics nearby then their counterparts. On the other hand, immigrants, were likely to attend cardiology visits in clinics with longer waiting times than non-immigrants. The gender interaction effects with distance and waiting were not significantly different from zero, neither were the coefficients of interactions of distance with immigrant status and waiting times with both income class and age.

Table 3 shows the estimates of the effect of a change in travel time, waiting time and a change in accessibility on hospital choice in the form of the elasticity of demand. Elasticities are calculated at the individual level and results are summarized with average elasticities. We followed the probability weighted elasticity model as proposed by Sivey (2012) [28]. The number of visits performed by each outpatient clinic are the associated weights.

**Table 3:** Average elasticities of demand for travel distance, waiting times and accessibility.

	Average elasticity of demand	SD
Ln(Travel time)—model 1	-2.042	0.356
Ln(Travel time)–—model 2	-1.753	0.294
Waiting time—model 1	-0.281	0.233
Waiting time—model 2	-0.335	0.263

The elasticities can be interpreted as the percentage change in demand associated with a 1% change in travel time and waiting time. Mean elasticities can be interpreted as the value of probability weighted elasticity averaged across all outpatient clinics. The results in Table 3 confirm that our regression models predict travel time has a much higher weight as a determinant of choice than waiting time. The direction of the elasticity coefficients confirm the results of a study on hospital choice for cataract surgery in the UK [28].

The mean travel time elasticities in model 1 and 2 were -2.042 and -1.753 respectively, whereas for waiting time, the elasticity in model 1 was -0.281 and -0.335 in model 2. For example, if we consider an outpatient clinic with a one-month outpatient waiting times which increases by 15 days (50%), then an elasticity of -0.281 predicts that admissions in that clinic will fall by 14.05%.

## Discussion

This paper provides an empirical investigation into the relationship between the choice of outpatient service and the patients’ willingness to travel and to wait. The patients’ socioeconomic conditions were also taken into consideration. The results show that patients have a strong preference for proximity (shorter travel time), rather than waiting times. All things being equal, travel time (proxy of distance) was the main discriminant, showing higher coefficients in all regression models and higher average elasticity of demand.

The elderly and patients in the lowest income class were more likely to choose closer clinics (physical proximity) compared to their counterparts. Often the limited travel means of these population subgroups restricts their choice, raising concerns over equity, especially vertical equity which refers to “the allocation of different resources for different levels of need” [29].

Inequalities concerning promptness and access to hospital and primary care services by vulnerable groups in Tuscany [29] seem to exist also with respect to outpatient care.

In fact, the statistically significant and positive interaction of waiting time with immigrant status of Model 2 suggests that immigrants, compared to non-immigrants, tend to receive care in clinics with longer waiting times.

Socioeconomic conditions are important determinates in patient choices regarding specialist care as already reported in analyses that found a significant pro-rich inequity in the use of outpatient services in Italy [30,31]. Factors that improve the traditional distance-decay model include the improved provision of patient transport (e.g., improving public transportation or providing subsidized transport) and improved planning and organizational capacity at the point of service. The latter refers to the harmonization of the booking systems at the regional level for routine visits and the inclusion of preferred booking options for deprived/fragile patients.

The findings present various limitations due to the quality of data and data availability which could improve the model and explanation of patient choice. Some of the limitations are due to the waiting time measurement. Current administrative data suffer from possible gaps in the harmonization of the different booking systems for outpatient services, which could lead to an underestimation of the effect.

The study could benefit from improved information on patient characteristics, such as education level, family circumstances (e.g., cohabitation status) and patient knowledge of alternative providers. Overall, the model only ascertained where patients had been treated and not whether they made an active choice [15]. Furthermore, we were not able to determine to what extent primary care physicians influenced these choices or whether a recommendation by family or friends influenced the decision. A proxy measure of GP influence on patient provider-choice could be to assess the extent to which informal cardiology outpatient referral networks between GP and the specialist exists. Thus the existence of a preferred cardiology outpatient clinic suggested by the GP could be identified using health administrative data on the natural links among patients, GPs and outpatient clinics based on the existing patient flow [32].

Despite these qualifiers and future improvements, one strength of this study is that administrative datasets were used at the individual level to highlight access issues that warrant further investigation. The study also offers a regional perspective on the geographical access to outpatient services, thus providing new evidence outside the traditional hospital care.

In conclusion, understanding how patients behave in the selection of providers can help managers and policy makers identify strategies to reduce patients' unmet needs [33] and equity gaps. These findings highlight the importance of examining and monitoring health equity, especially with regard to the socioeconomic disaggregation of statistical data (vertical equity) in order to identify groups or areas in need, and may be useful for implementing quality improvements [29]. Lastly, the results contribute to the debate on the failure of quasi-market mechanisms where the policy of patient choice was modelled on the premises of rational (well-informed) individuals [34]. However, in the healthcare sector, the information asymmetry between user and provider constrains choice especially for lower socio-economic groups.

## Supporting information

S1 File This is the dataset. (TXT)

## References

[1] Brekke KR, Gravelle H, Siciliani L, Straume OR. Patient Choice, Mobility and Competition Among Health Care Providers. Dev Health Econ Public Policy. 2014;12. 10.1007/978-88-470-5480-6_1 24864380

[2] Dixon A, Robertson R, Bal R. The experience of implementing choice at point of referral: A comparison of the Netherlands and England. Heal Econ Policy Law. 2010;5(3):295–317. 10.1017/S1744133110000058 20462469

[3] France G, Taroni F. The Evolution of Health-Policy Making in Italy. J Health Polit Policy Law. 2005;30(1–2):169–87. 15943392 10.1215/03616878-30-1-2-169

[4] Magnussen J, Vrangbaek K, Saltman RB. Nordic Health Care Systems: Recent reforms and current policy challenges. Nordic Health Care Systems. 2009. 1–344 p.

[5] Shah J, Dickinson CL. Establishing which factors patients value when selecting urology outpatient care. Br J Med Surg Urol. 2010;3(1):25–9.

[6] Coulter A. Do patients want a choice and does it work? BMJ. 2010;341. 10.1136/bmj.c4989 20947576

[7] Cooper Z, Gibbons S, Jones S, Mcguire A. Does hospital competition save lives? Evidence from the English NHS patient choice reforms. Econ J. 2011;121(554):228–60. 10.1111/j.1468-0297.2011.02449.x PMC4373154 25821239

[8] Bevan GA, Evans; Nuti S. Reputations Count: Why benchmarking performance is improving health care across the world. Heal Econ Policy Law—Forthcom. 10.1017/S1744133117000561 29547363

[9] Berkowitz EN, Flexner WA. The market for health care services: Is there a non-traditional consumer? J Health Care Mark. 1981;1:25–34. 10251474

[10] Boscarino J, Stebier SR. Hospital shopping and consumer choice. J Health Care Mark. 1982;(2):23–5. 10256047

[11] Wolinsky FD, Kurz RS. How the public chooses and views hospitals. Hosp Heal Serv Adm. 1984;29:58–67. 10268661

[12] Lane PM, Lindquist JD. Hospital choice: A summary of the key empirical and hypothetical findings of the 1980s In: In Cooper P (Ed), Health care marketing. third edit Maryland: Aspen Publishers, Inc; 1994. 10303067

[13] Balia S, Brau R, Marrocu E. What Drives Patient Mobility Across Italian Regions? Evidence from Hospital Discharge Data. Dev Health Econ Public Policy. 2014;12:133–54. 24864385 10.1007/978-88-470-5480-6_6

[14] Toscana Re. Sanità e salute in Toscana: dati sintetici 2015–2016. Regione Toscana Website. p. http://www.regione.toscana.it/sanita-e-salute/stat.

[15] Aggarwal A, Lewis D, Mason M, Sullivan R, van der Meulen J. Patient Mobility for Elective Secondary Health Care Services in Response to Patient Choice Policies: A Systematic Review. Med care Res Rev. 2016; 10.1177/1077558716654631 PMC5502904 27357394

[16] Exworthy M, Peckham S. Access, choice and travel: Implications for health policy. Soc Policy Adm. 2006;40(3):267–87.

[17] Penchansky R, Thomas JW. The concept of access: definition and relationship to consumer satisfaction. Med Care. 1981;19(2):127–40. 7206846 10.1097/00005650-198102000-00001

[18] Nuti S, Vainieri M. Strategies and tools to manage variation in regional governance systems In: Stukel TA, Johnson CCA, editors. Medical Practice Variations. Springer; 2014 p. 1–40.

[19] Gesler MW, Meade MS. Locational and population factors in health care-seeking behavior in Savannah, Georgia In: In De Friese GI, Ricketts TC, and Stein SJ (Eds), Methodological advances in health services research. Ann Arbor: 1989. PMC1065514 3403277

[20] Fattore G, Mariotti G, Rebba V. Waiting Time Policies in the Health Sector: What Works? Vol. 3001, Waiting Time Policies in the Health Sector: What Works? 2013. 1–328 p.

[21] Ferré F, Giulio A, Valerio L, Longhi S, Lazzari A, Fattore G, et al Health System Review: Italy. Health Syst Transit. 2014;16(4):1–168. 25471543

[22] Ministero della salute. Piano Nazionale di Governo delle Liste di Attesa. 2012.

[23] Briggs DJ, Gulliver J, Fecht D, Vienneau DM. Dasymetric modelling of small-area population distribution using land cover and light emissions data. Remote Sens Environ. 2007;108(4):451–66.

[24] Boo G, Fabrikant SI, Leyk S. a Novel Approach To Veterinary Spatial Epidemiology: Dasymetric Refinement of the Swiss Dog Tumor Registry Data. ISPRS Ann Photogramm Remote Sens Spat Inf Sci. 2015;II-3/W5(SEPTEMBER):263–9.

[25] Arne RisaHole. Modelling heterogeneity in patients’ preferences for the attributes of a general practitioner appointment. J Health Econ. 2008;27(4):1078–94. 10.1016/j.jhealeco.2007.11.006 18179837

[26] Arne RisaHole. MIXLOGIT: Stata module to fit mixed logit models by using maximum simulated likelihood. EconPapers. 2016;

[27] ARS T. Rapporto Crisi economica, stato di salute e ricorso ai servizi in Toscana. 2013;

[28] Sivey P. The effect of waiting time and distance on hospital choice for English cataract patients. Health Econ. 2012;21:444–56. 10.1002/hec.1720 21384464

[29] Barsanti S, Nuti S. The equity lens in the health care performance evaluation system. Int J Heal Plann Manag. 2014;29(3):e233–46. 10.1002/hpm.2195 23722829

[30] Masseria C, Giannoni M. Equity in access to health care in Italy: A disease-based approach. Eur J Public Health. 2010;20(5):504–10. 10.1093/eurpub/ckq029 20504952

[31] Glorioso V, Subramanian S V. Equity in access to health care services in Italy. Health Serv Res. 2014;49(3):950–70. 10.1111/1475-6773.12128 24949515 PMC4231580

[32] Stukel TA, Glazier RH, Schultz SE, Guan J, Zagorski BM, Gozdyra P, et al Multispecialty physician networks in Ontario. Open Med. 2013;7(2):1–16. PMC3863751 24348884

[33] Akinci F, Esatoglu AE, Tengilimoglu D, Parsons A. Hospital choice factors: a case study in Turkey. Health Mark Q. 2005;22(1):3–19. 10.1300/j026v22n01_02 15914381

[34] Fotaki M. Patient choice in healthcare in England and Sweden: From quasi-market and back to market? A comparative analysis of failure in unlearning. Public Adm. 2007;85(4):1059–75.

